# RF-Based Location Using Interpolation Functions to Reduce Fingerprint Mapping

**DOI:** 10.3390/s151027322

**Published:** 2015-10-27

**Authors:** Santiago Ezpeleta, José M. Claver, Juan J. Pérez-Solano, José V. Martí

**Affiliations:** 1 Departament d’Informàtica, Universitat de València, Avd. de la Universitat, Burjassot 46100, Spain; E-Mails: Santiago.Ezpeleta@uv.es (S.E.); juan.j.perez@uv.es (J.J.P.-S.); 2 Department of Computer Science and Engineering, Universitat Jaume I, Campus Riu Sec, Castellón 12071, Spain; E-Mail: vmarti@uji.es

**Keywords:** RF-Location, finger-printing, interpolation, 802.15.4 networks

## Abstract

Indoor RF-based localization using fingerprint mapping requires an initial training step, which represents a time consuming process. This location methodology needs a database conformed with RSSI (Radio Signal Strength Indicator) measures from the communication transceivers taken at specific locations within the localization area. But, the real world localization environment is dynamic and it is necessary to rebuild the fingerprint database when some environmental changes are made. This paper explores the use of different interpolation functions to complete the fingerprint mapping needed to achieve the sought accuracy, thereby reducing the effort in the training step. Also, different distributions of test maps and reference points have been evaluated, showing the validity of this proposal and necessary trade-offs. Results reported show that the same or similar localization accuracy can be achieved even when only 50% of the initial fingerprint reference points are taken.

## 1. Introduction

Recent advances in telecommunications technology have provided a rapid development of mobile and autonomous devices with low cost and power consumption, which can perform multiple functionalities. Wireless Sensor Networks (WSN) are part of this phenomenon and can be found nowadays deployed everywhere in multiple applications and monitoring multiple parameters.

The location information is extremely helpful in the development of new context-aware applications, where it is used to provide a wide variety of services to the final user. In outdoor scenarios, Global Positioning System (GPS) [1] represents the most important technology that can be used to locate a node in a certain area. Another technology that can be combined with the previous one is the mobile phones Cell-ID, but its accuracy is not as high as in the GPS case, with a precision that is in the order of hundreds of meters to kilometers, depending on the cells size.

In indoor environments these technologies can not be applied because the GPS signal is not available and the coverage of the mobile phones sometimes is poor. For these scenarios, communications systems based on radio frequency (RF) technologies, such as WiFi (used in the deployment of WLAN) and the IEEE 802.15.4 standard (very popular in the implementation of WSN), are used for location and tracking purposes.

Many RF-based strategies have been proposed in the last decade for indoor location and tracking. Analytic location methods are based on the signal strength received to compute the distance to the transmitter applying an attenuation factor to the original signal by using the following expression:

$$P=A*dist^{-n}$$

where *P* is the strength of the received signal, *A* is the strength of the original transmitted signal, dist is the distance between transmitter and receiver, and *n* is the attenuation coefficient in the transmission medium. Several systems, e.g., the integrated in micro-controllers as the CC2431 by Texas Instruments (called Location Engine) [2], calculate the distance between every beacon and the transmitter using the original signal strength and the propagation coefficient in the medium. Thus, with three or more beacons the transmitter’s position can be calculated using triangulation. The use of this method outdoors brings acceptable results, since the signal propagation is close to ideal case. However, in indoor applications, the received signal strength indicator (RSSI) can be affected and altered by reflections from walls, floors, furniture and ceilings. Since it is very difficult to resolve analytically this propagation model, it is possible to improve the accuracy of the analytic methods using fingerprinting techniques as it is described in [3,4,5,6]. It implies an initial training step to collect RSSI measures in some chosen reference points in the considered area to create a database. These RSSI samples are used in the location phase to estimate the node real position. For this purpose, pattern recognition techniques to compare the measures collected during the localization phase with the values stored in the training database are applied.

However, fingerprinting methods have some drawbacks because the initial construction of the database needs a considerable amount of time, and in case of modifications, it is necessary to rebuild it. Additionally, during the location phase the involved algorithms need considerable amount of data, large amounts of memory and computation resources to carry out the location estimation in real time. There are some other alternative algorithms that modify the original fingerprinting method using probabilistic techniques, pattern recognition algorithms, spatial filters, diversity of signal strength and channels, different number of beacons, *etc.* to improve the localization accuracy. Furthermore, as the real world localization environment is dynamic, it is usually required to rebuild the fingerprint database when some environmental changes are made. Adding more references points with the interpolation method to the fingerprint database saves time and improves localization accuracy.

In this paper, we analyze the location accuracy that can be achieved depending on the amount of information saved in the RSSI database. For this purpose, we study the influence of considering a different number of fingerprint training points and the use of interpolation functions to reduce the initial effort during the training phase. In the article, different interpolation functions are compared to estimate RSSI values in new positions not integrated in the training step. To complete the study, different distributions of test maps and densities have been evaluated. This technique can be combined with the selection of more significant signal strength levels and frequency channels to optimize the size of the database [7].

The remainder of this paper is organized as follows. In the next section the existing RF location and tracking techniques proposed until now for indoors are described. Section 3 is devoted to reviewing the interpolation methods and show their utility in other widely studied problems. Section 4 describes the start methodology used for RF location and how the use of interpolation methods can improve the way this one is carried out. The experimental results showing the advantages of using our approach are presented in Section 5. Finally, concluding remarks are given in Section 6.

## 2. Related Work

In the bibliography, there are many RF-based methods that have been proposed to perform indoor location and tracking. One of the most precise approaches has been obtained combining radio signal strength indicator (RSSI) measures and fingerprinting. As it was aforementioned, fingerprinting methods require a first training stage, in which the RSSI values from the network beacons or base stations deployed are collected positioning the testing node at different reference points within the considered area. During the location stage, the method applies a pattern recognition algorithm to find in the database the point that better match the RSSI values taken at the current position of the node.

The first proposed approach using the RF fingerprinting method was RADAR [8], which uses the Wi-Fi signal from WLANs. Another example is MoteTrack [9], which uses the radio signal of IEEE 802.15.4 transceivers integrated in WSNs motes. Instead of the centralized algorithm implemented by RADAR, MoteTrack proposes a distributed approach that provides a more robust and reliable algorithm. Another example is the work presented in [10] that outperforms the results presented in [8]. In this case, a RF-map learned during the training phase improves the precision of the algorithm [10] and in this paper is also shown how the presence of furniture, objects, walls, *etc*. makes ineffective the theoretical $1/r^{2}$ attenuation model.

Probabilistic estimation methodologies for increasing the accuracy of the location algorithm have been proposed in [11,12]. Reference [13] compares deterministic versus probabilistic location methods and shows that increasing the information provided for each point in the training phase allows the use of more sophisticated approaches at the expense of a more tedious process in the fingerprint configuration.

Euclidean and Manhattan distances have been used in deterministic location methods [8,9,11]. The Euclidean metric is the most widely spread, and its results can be improved taking the most accurate information in the training phase [14,15,16], selecting the signal strength level [13], or introducing fuzzy logic [17]. Other methods of indoor location combine simultaneously RSSI with additional parameters obtained from physical sensors (movement, acceleration, *etc*.) to reduce time and cost in the calibration phase [18].

The effects of the number, distance and distribution of sampling points employed in the RF-based fingerprint matching are analyzed in [19], but no interpolation methodologies are considered in this study. A recent approach using interpolation models to improve results in positioning based on the attenuation of the signal strength [20] shows a reduction of the database size and the time spend in the training phase. In this approach, environmental conditions of the scenario are considered for the estimation model used, as in [21].

In all the previous references, only one frequency channel is normally used in the radio packet transmission. A first exception to this rule is presented in [22], where a very large number of carriers (500 channels) are taken into account for implementing an indoor location method based on cellular telephony. In this reference the localization is performed with a classification algorithm that includes support vector machine techniques. The algorithm involves a long training phase and presents a high computational cost, but it achieves accurate location results. A recent variant of these techniques presented in [7,16] takes advantage of the flexibility of the current IEEE 802.15.4 transceivers, in terms of the selection of the frequency channels and RSSI signal strength levels, to increase the number of RSSI measures taken at each reference point. With the increment of the total amount of information that is available from different channels and signal strength levels more accurate location estimations can be achieved. A more intelligent way of combining the information of different frequency channels and signal strength levels is to select only those channels and levels that provide more accurate information [7]. Thus the algorithm rejects the channels and power levels that are more affected by noise or interference coming from other technologies sharing the same frequency bands. An alternative way of selecting the information collected during the training phase implies the rejection of experimental RSSI values corresponding to signal levels that are too weak. The idea proposed in [23], and named as Short Is Better (SIB), implies the elimination of those RSSI measures that are very low and fall below a certain threshold. The rationale behind this approach is to remove information that comes from weak signals because it might be more easily altered by the environmental noise.

In this paper, it is analyzed the influence of the number of reference points taken during the training phase in the location precision. In addition, different interpolation functions are evaluated to estimate new RSSI values and construct the RSSI fingerprint database from a reduced number of experimental reference points.

## 3. Interpolation Methods

Interpolation is a mathematical tool that can estimate the value of a function at a certain point using other available values of this function at different points. Interpolation models are basically deterministic, because they treat the function outputs as fixed values. Several mathematical models can be applied depending on the required accuracy and complexity. As a first approach, the estimation can be performed combining in some manner the known function values. Since the known points closer to the new estimated function value should have a greater influence in the interpolation, the known values have to be combined by using a weight function, which should be a positive decreasing function of the distance. This type of interpolation models are usually known as kernel smoothers and one common example is the inverse distance weighting (IDW) [24] interpolation, where the weight function is a simple inverse power function in *ℜ* expressed as:

(1) $$w\left(x\right)=x^{-a}$$

where the constant *a* is a positive value. Another example of weight function is the exponential:

(2) $$w\left(x\right)=e^{-ax}$$

where again *a* is a positive value.

The final expression of the interpolation function using these weights is:

(3) $$\hat{y}\left(x_{0}\right)=\frac{\sum_{j}w\left(d_{j}\right)\cdot y\left(x_{j}\right)}{\sum_{j}w\left(d_{j}\right)}$$

being $y(x_{j})$ the set of known or measured values of the function, and $d_{j}$ the distance between these points ($x_{j}$) and the new estimated point ($x_{0}$) in the *x* axis.

Other types of interpolation models are those based on polynomial functions. In this case, it is intended that the original curve can be modeled using a polynomial function of a certain order. The simpler case implies the use of a linear regression that can be resolved using the well-known least squares procedure for obtaining the slope and the intercept. The local application of the linear regression allows the adjustment of more complex functions defining a diameter in which the linear interpolation is valid. Kernel smoothing weight functions can be introduced in the least squares calculation to cancel the contribution of points that are out of this local interval, reducing their importance when they are farther from the central point that is being estimated. Following the same procedure at every estimated point, the continuity of the fitting curve can be preserved. For functions with abrupt changes, the local interpolation can be performed considering polynomials of higher orders.

With the previous models, continuous interpolation surfaces can be achieved. However, sometimes, it is not enough to ensure the continuity of the model, but it is also required the smoothness at every point. This requirement can be met using radial basis function (RBF) interpolators [24]. These basis functions are radially symmetric around the origin and decline towards zero as we move away. Some examples of radial basis functions calculated at a point *s* in ${ℜ}^{2}$ are:

- The Euclidean distance linear basis function.
  (4) f(s)=∥s∥
- The multiquadratic function.
  (5) $$f\left(s\right)=\sqrt{\left(1+∥s{∥}^{2}\right)}$$
- The thin plate spline function.
  (6) $$f\left(s\right)={∥s∥}^{2}\log\left(∥s∥\right)$$
- Polyharmonic spline functions.
  (7) $$f\left(s\right)=\left\{\begin{array}{cc} {∥s∥}^{n}\log\left(∥s∥\right) & \text{if }n\text{ is an even integer}\\ {∥s∥}^{n} & \text{otherwise} \end{array}\right.$$

In the last case of polyharmonic spline functions, there are some combinations of functions and powers that are not suitable (e.g., ${∥s∥}^{2}$), because the interpolation equations might not have a solution. Using this interpolation method, the origin of one basis function is placed at every position where a known function value is available. The model is achieved using a weighted combination of all the basis functions. The calculation of the weights is made in such a way that ensures the equality between the interpolation results and the initial known values at the origins of the radial basis functions. The calculation of the weights can be carried out solving the following system of linear equations:

(8) $$y_{i}\left(s_{i}\right)=\sum_{j=1}^{n}w_{j}f_{j}\left(s_{i}\right),\;\;\;\;i=1,\ldots ,n$$

where $y_{i}\left(s_{i}\right)$ is the set of known values used in the interpolation, $s_{i}$ are the points where the known values were taken, $w_{j}$ are the weights and $f_{j}\left(s\right)$ are the radial basis functions, each one centered at a different $s_{i}$ point. The main features that are provided by the combination of these radial basis functions are: (a) the smoothness of the resulting interpolated surface, since it comes from the addition of smooth functions, and (b) the accuracy against the real underlying model, because the interpolation results are equal to the known function values at the origin of the radial basis functions.

These interpolation methods have been successfully applied to the estimation of dense radio environment maps from a reduced number of reference points in which measurements of the RSSI are taken [25,26,27]. Using these techniques the fingerprinting database can be built with less effort because a much lower number of reference points are sampled and the rest of the radio map is estimated using interpolation. These techniques have been applied to the construction of both indoor and outdoor radio maps for technologies such as: GSM mobile phone signal and wireless LAN based on 802.11 technologies [27]. Results shown in references [25,26] demonstrate that the localization accuracy of the fingerprinting algorithms with interpolated radio databases, using very few measured reference points in 802.11 networks, is practically the same to the cases in which the database comprises a great number of measured reference points. In this article, this method is applied to IEEE 802.15.4 networks that allow the selection of multiple frequency channels and radio signal power transmission levels. Thus, the location algorithm can combine the information from multiple interpolated radio maps corresponding to different frequency channels and power levels.

## 4. Proposed RF-Based Localization

This section is organized in two parts. Firstly, the bases of RF-based location using fingerprint of signal strength, are described. In particular, we focus our description on the methodology followed in [16], because this is the reference work used to compare with our proposal. In the second part, some ideas to reduce the time spent collecting the information data needed in the location process are presented. The key element for the success of this approach is the use of interpolation functions.

### 4.1. Methodology

The location proposal presented in [16] takes advantage of the flexibility of current IEEE 802.15.4 transceivers to configure the frequency channel and signal strength levels for radio packet transmissions. With the increase in the number of RSSI samples and combining values from different channels and signal power levels more accurate locations can be estimated. The method runs in two phases: (1) Training; and (2) Location estimation.

**1. Training.** In the training stage, RSSI measures in different positions distributed uniformly through the entire location scenario are taken. So, for every reference point (i,j), the RSSI value (*p*) of different channels (*c*) and different beacons (*b*) deployed in the location scenario are sampled and save in the database. To avoid measuring errors, ten samples (five in each direction) at every reference point are taken and saved. The transmission is started at the mobile node that sends a packet received by the network beacon. Then, the beacon replies sending a new packet in the opposite direction. This packet exchange is repeated 5 times and a total of 10 packets are transmitted (5 in each direction). So, at the end 10 RSSI values are sampled, 5 measuring the RSSI received at the network beacon and another 5 RSSI values taken at the mobile sensor. The system sends packets in both directions to check the difference between the RSSI values measured at the mobile node and at the beacons.

Thus, a vector of p×c×b components is formed and saved in a centralized database for each reference point. Once the whole scenario is measured, some methods for reducing the database size can be applied. Two possible alternatives are: (a) to compute the mean of the five packets or (b) to eliminate the maximum and minimum values and save the mean of the three remaining values.

**2. Location estimation.** After the initial training step, the mobile sensor can be located in the tested scenario. The method used in this work is a variation of the k-nearest neighbors (KNN) algorithm, which selects the k-nearest samples in the radio map with a higher similarity to the current measure. The algorithm takes into account all the information saved in the database that includes different channels and power levels. A detailed description of the location algorithm can be found in [16].

### 4.2. Proposals for Improving the Location

Currently, the devices conforming a WSN use the radio standard IEEE 802.14.5 as transmitting technology and RF transceivers, allowing the selection of different frequency channels and radio signal strengths. Using more channels the method can increase the information provided for each reference point with respect to the use of only one channel, which is typical for WLAN systems. This process requires larger time to synchronize the network beacons and the mobile device in the reference point, but this information increase allows more accurate location algorithms [16].

When there is a significant difference between the frequency of two signals, a different behavior regarding obstacles and walls occurs. For example, one frequency can border an obstacle, while the another can not traverse it, or one frequency is highly attenuated by a wall, while another passes through the wall with little attenuation. However, the difference in frequency between the channels used in WSN is small. So, there are not significant or appreciable differences for different channels. However, different channels can be affected by interferences with other RF signals present in the transmitting medium, such as: WIFI, Bluetooth, *etc*. As a consequence, some packets can be lost or the RSSI value received by the location node can be incorrect. So, introducing channel diversity, the errors affecting the measures in a noisy channel could be reduced.

Marti shows in [16] that the location accuracy depends on the level of the radio signal strength. Weak signals have problems for distant points due to the possibility of packet loses, while they provide important information for nearby points. In contrast, strong signals can provide little information for points near to a network beacon, but are very useful for distant points. Thus, the combination of different signal strengths covers different situations in a location scenario, as it is showed in the proposal .

The training step of the fingerprinting method requires a great effort because the mobile sensor has to be placed at every reference point of the location scenario. To reduce this initial effort, the database can be initially constituted using a reduced number of experimental reference points in the scene and it can be extended afterwards, generating the rest of the RSSI values with interpolation functions. This methodology could be combined with the selection of different channels and power levels. So, the database in this case is built including experimental RSSI values for each frequency channel and power level at a reduced number of reference points. Once the experimental measures are completed, the database can be generated using the interpolation functions to form a radio map for every frequency channel and power level in an independent way. Consequently, the final database will contain independent radio maps covering the whole location area for each combination of frequency channel and power level. Information from different maps is combined later by the location algorithm, as it was exposed in the previous Section 4.1, to increase the diversity of channels and power levels and to improve the robustness against interferences and measure errors.

In the next section, various experimental tests have been carried out to evaluate all the previuos proposed algorithms and to determine the influence of the different solutions in the location precision.

## 5. Experimental Results

This section presents the experiments that we have carried out to check the location accuracy of the algorithm proposed in Section 4. During the tests we have used an experimental database of RSSI values taken in an indoor area. The objectives of these experiments are twofold. On the one hand, the first objective is to evaluate the error introduced in the database values when the number of reference points is reduced during the training phase. The points not measured are estimated using the interpolations functions seen in Section 4. On the other hand, it is also important to evaluate the precision achieved in the location algorithm when the database includes interpolated RSSI values.

### 5.1. Case Study and Configuration

**Figure 1:**
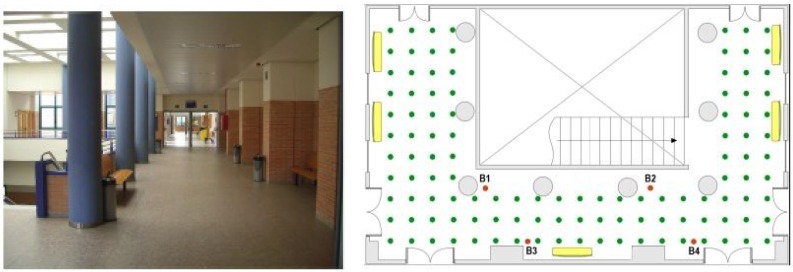
Photo and map of the room where the system was deployed.

This subsection describes the indoor scenario used in our experiments. Figure 1 shows the map of the room where the network was deployed. The scenario covers an area of 11 × 19 m and it includes some building elements such as: reinforced concrete columns, stair well with steel rails, metal doors, irregular walls and latticework in the ceiling, elements that substantially disrupt the propagation of signals and complicate the location estimation. There were four beacons placed at positions marked as: B1, B2, B3, and B4 and the mobile sensor was placed at each reference point of the grid marked with a green dot (see Figure 1). The grid comprised 113 reference points, located one meter apart from each other. During the training phase, the mobile node was placed at every reference point of the grid to exchange data packets with each beacon using six different channels: CH11, CH13, CH16, CH19, CH22, CH26 (with carrier frequencies (MHz): 2405, 2415, 2430, 2445, 2460, and 2480, respectively), and four power levels: P3, P19, P95, P255 (gains (dBm): −25.2, −5.7, −0.4, and 0.6, respectively). Since ten data packets were exchanged with each beacon (5 in each direction) for every different combination of channel and power level, a total amount of 960 RSSI values were measured and saved in the database at each reference point.

### 5.2. Analysis of the Database Interpolation

The fingerprinting training step represents the main drawback of these localization methods. To lighten this initial effort, the number of reference points can be decreased, but at the expense of decreasing the density of information available during the localization phase. One way to reduce the number of experimental measures required during the training, without decreasing the number of reference points in the database, is to estimate a part of the RSSI values using interpolation functions. In this subsection, the feasibility of this method for different distributions and densities of reference points is evaluated.

**Figure 2:**
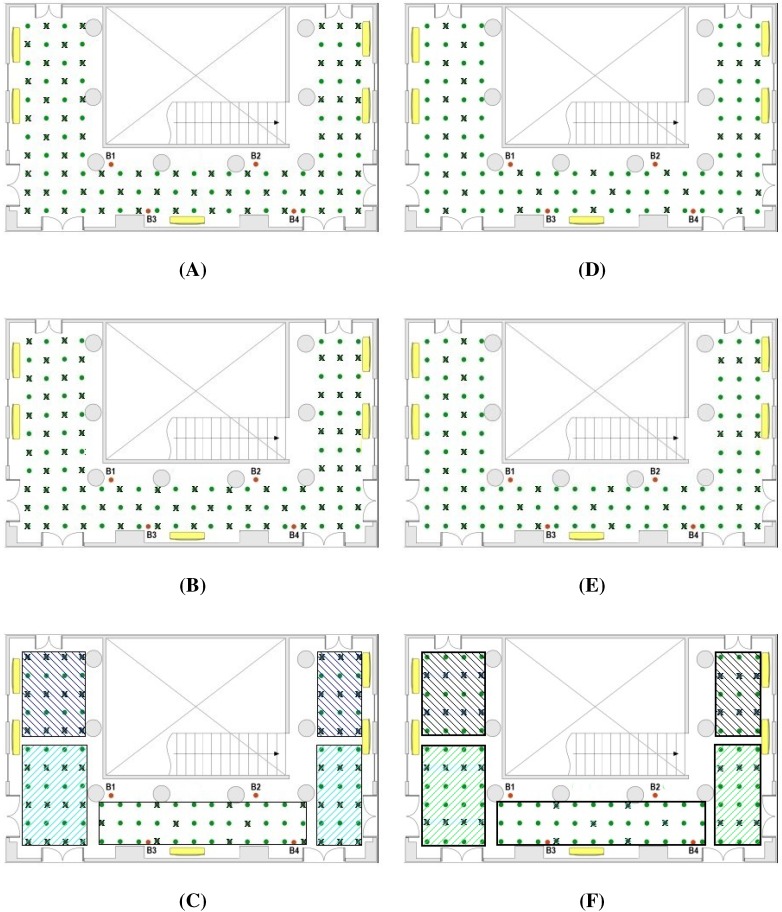
Map of the testing scenario with: ( left) 50% of reference points for three different distributions: (**A**) Uniform; (**B**) Uniform with the complementary points of case A; (**C**) Non-uniform by zones; and (right) 25% of reference points for other three distributions: (**D**) Uniform; (**E**) Uniform with alternative points of case D; (**F**) Non-uniform by zones. The “×” denotes the reference points, whereas the green dots are the interpolated ones.

For testing the accuracy of this method, the measured RSSI values in the database are compared with the estimated values obtained from the interpolation functions. To this end, the initial database, which includes all the testing points shown in the previous section, is reduced removing the RSSI values corresponding to 50% of the total amount of reference points. Using the remaining RSSI values and applying the RBF interpolation functions, the RSSI values for the removed reference points are estimated. Figure 2 depicts two possible distributions of reference points, where the original reference points are marked by a ×, and the interpolated ones are represented only by a dot. Figure 2A,B show a uniform distribution in all the location area. Nevertheless, Figure 2C exhibits an non-uniform distribution by zones, where the global density of reference points is of 50%, but the density in each zone varies from 25% to 60%. The idea is that RF signals have better quality for locations near to the beacons, and poor quality for locations far from the beacons. Hence, for zones near to the centroid of the beacons a low density of reference points (25%) is used, while for zones away from the centroid of the beacons the density of reference points is increased (until 60%).

**Figure 3:**
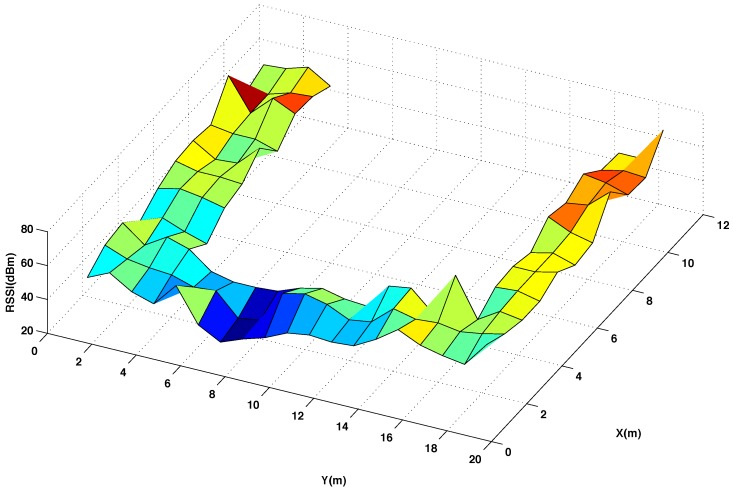
Radio map of RSSI for the original reference points in the testing area. Using channel CH11 and power level P3.

**Figure 4:**
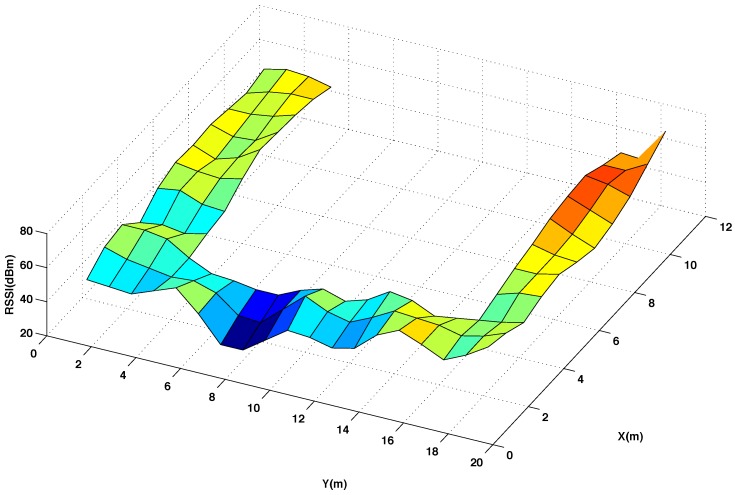
Radio map of RSSI for 50% original and 50% interpolated (using the thin spline function) reference points in the testing area. Using channel CH11 and power level P3.

Figure 3 and Figure 4 show graphically the radio map of RSSI values measured at every reference point of the testing area, with and without interpolated points, respectively. In Figure 3 and Figure 4 the RSSI values are the average of 5 five measures taken at the mobile sensor when the beacon 4 transmits 5 packets using channel CH11 and power level P3. Comparing the two radio maps, it can be noticed that the interpolated version smooths out the original surface and produces accurate results for most of the interpolated points, providing that the changes in the RSSI values between neighbouring points are not abrupt.

**Figure 5:**
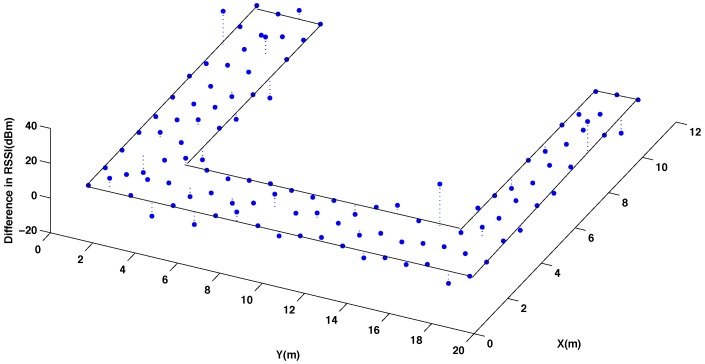
Difference in the radio map of RSSI for 50% original and 50% interpolated (using the thin spline function) reference points in the testing area. Using channel CH11 and power level P3.

Figure 5 presents the difference of the RSSI values between these two radio maps. As it can be seen, higher errors are mostly concentrated in few points where the original map presents abrupt changes. To quantify the total error committed using the interpolation, it has been calculated the average percentage of error between the measured and the interpolated RSSI values considering all the reference points of the testing room, as it is represented in Figure 2A.

Table 1 summarizes the results obtained with the four different interpolation functions. As a conclusion, we can see that the best result is achieved for the thin spline function since the obtained radio map has the lower difference with respect to the original one. This is due to fact that the thin function approximates better the real shape of the RSSI behavior measured in dBm.

**Table 1:** Average error between the measured and estimated RSSI (in percentage), when 50% of the reference points are interpolated.

	Thin	Euclidean	Multiquadratic	Polyharmonic (n = 4)
Difference in RSSI values (in %)	6.30 %	7.80%	7.80%	9.80%

### 5.3. Analysis of Location Accuracy

This subsection presents a study about the location accuracy obtained for different grades of the experimental database size. This evaluation allows to establish a trade off between the number of experimental reference points taken during the training step and the location accuracy that can be achieved. In the analysis, the same testing network and localization area presented in Figure 2 has been used. As a first approach, the analysis focuses on the case where 50% of the RSSI values are interpolated, as it is represented in Figure 2. In this figure, the points marked with the × are the original reference points and the point marked with a dot are interpolated. Once the new database is completed with the interpolated RSSI values, the location algorithm presented in Section 4.1 is applied. The location algorithm is executed using a second data set of experimental RRSI values taken by the mobile node at the same positions of the testing grid in a second round of experimental measures. The analysis includes the application of the location algorithm presented in reference [16] with three different densities of reference points:

- 100% of experimental reference points and without any interpolated point, this case produces an average location error of 2.02 m with an standard deviation of 1.88 m,
- 50% of the experimental reference points and the other 50% interpolated in three cases: case A with a configuration shown in the scheme of Figure 2A; case B, complementary of case A; and case C, choosing the reference points by zones according to their distance to beacons (Figure 2C). The main difference between the A and B distributions is that in the former most of the points blocked by obstacles in their line of sight to the beacons are included as reference points. In contrast, the B distribution includes hardly any blocked point and they are inserted in the database as interpolated points.
- 25% of experimental reference points and 75% interpolated in three cases: case D with a configuration shown in the scheme of Figure 2D; case E, complementary of case D; and case F, choosing the reference points by zones according to their distance to beacons (Figure 2F).

Table 2 presents the mean location error and the standard deviation for densities with 50% and 25% of reference points.

**Table 2:** Average localization accuracy with different interpolation functions (thin spline, euclidean, multiquadratic and polyharmonic with n = 4) and percentage of reference points: 50% and 25%. In both cases, three different selections of the interpolated points are considered.

		Thin	Eucl	Multiqua	Polyhar n = 4
case A (50%)	mean error (m)	2.02	2.09	2.12	2.22
	standard deviation (m)	1.90	2.51	2.27	2.42
case B (50%)	mean error (m)	2.50	2.38	2.52	3.10
	standard deviation (m)	2.60	2.53	2.66	3.25
case C (50%)	mean error (m)	2.16	2.28	2.42	2.53
	standard deviation (m)	2.06	2.18	2.40	2.55
case D (25%)	mean error (m)	3.04	3.12	3.26	3.34
	standard deviation (m)	3.18	3.25	3.45	3.59
case E (25%)	mean error (m)	2.98	3.06	3.22	3.52
	standard deviation (m)	2.63	3.16	3.21	3.56
case F (25%)	mean error (m)	3.18	3.33	3.42	3.56
	standard deviation (m)	3.26	3.42	3.53	3.66

A first look at the Table 2 reveals that the algorithm accuracy does not decrease significantly when the database includes 50% of interpolated points. So, the influence of the interpolation in the algorithm performance is practically negligible for this percentage of reference points.

The set for reference points selected has an influence in the average error obtained. Thus, for the case A the average location accuracy is practically equal to the case with 100% of the reference points, whereas for the case B, using the complementary reference points, the average location accuracy is clearly worse. This behavior is the same for all the interpolation functions, therefore there is a direct influence on the quality of the selected reference points. The difference between the results of case A and B can be due to the number of points blocked by obstacles taken in the database as reference or interpolated points. Thus, the A distribution includes most of the blocked points as reference points, whereas in the B distribution they are interpolated. Another issue that can be considered is the distance between the reference points and the beacons. This matter is evaluated using the case C, which represents a non-uniform distribution divided in zones with different densities of reference points. In case C, the global distribution of reference points is 50%, but depending of the distance of the zone to the beacons, the density of reference points goes from 25% (near to beacons) to 60% (far from beacons). Table 2 shows that this distribution could be a good option because of the average location accuracy has an intermediate value between the case A and B.

This is an important result because it means that with half the number of experimental points we can achieve the same location precision. However, when the number of reference points that are interpolated grows too much, *i.e.* the case D with 25% of reference points, the precision of the location algorithm falls, achieving a mean error around 3 m. The same behavior can be observed in the two other distributions E and F.

In the case of 50%, it can be noticed that the best location precision is obtained with the thin spline interpolation function. This fact is in accordance with the results of the previous subsection, because this is the function that produces a lower difference between the interpolated RSSI values and the experimental measures. So, it is reasonable that this interpolation function provides the highest location accuracy.

**Figure 6:**
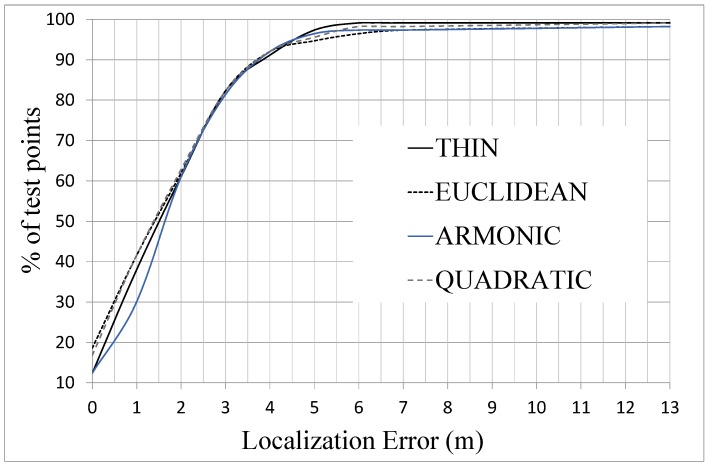
Percentage of test points by localization error for case A of Table 2 using different interpolation functions.

Additionally, in Figure 6 it is shown the percentage of test points that produce a localization error that is lower than a certain value. The main result drawn from this graph is that 80% of test points produce an error lower than 3 m and only a 10% of test points are located with an error higher than 4 m. The differences between the interpolation functions are not very important, although it can be seen that the thin function is closer to 100% of test points when the localization error exceeds 4 m.

**Figure 7:**
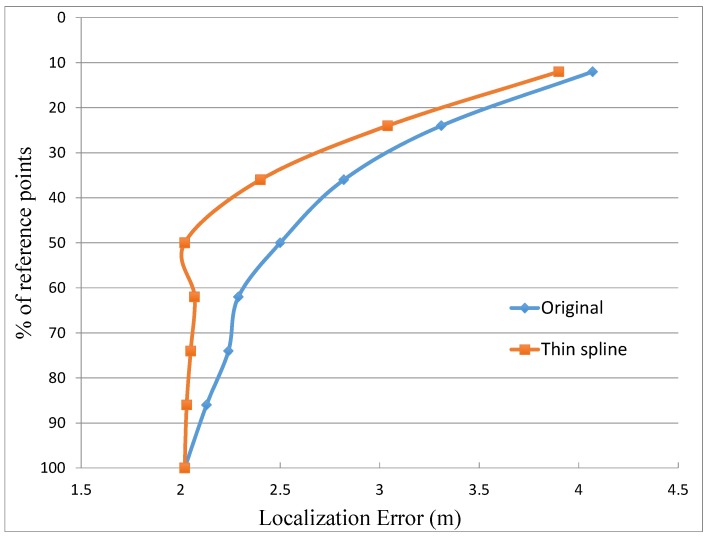
Percentage of reference points included in the database against the localization error for the original case without interpolation and using thin spline interpolation.

Another important issue that must be considered is the dependence of the location accuracy on the total amount of reference points. This matter has been evaluated taking eight different percentages of reference points, spaced in the interval from 100% to 10% of the total amount of reference points. For every percentage, the original case without interpolation and the one applying the “Thin spline” interpolation have been applied. Figure 7 presents the obtained results, which shows clearly how the case without interpolation rapidly worsens when the number of reference points decreases. Thus, the elimination of only around 10% of the total amount of reference points makes this case to lose more than 10% of the location accuracy. In contrast, the case with interpolation keeps a good precision even with percentages of reference points in the order of 50%. In this interval, from 100% to 50%, the case with interpolation produces results with a degradation lower than 10% of the initial precision. Only when the limit of 50% of reference points is reached, the case with interpolation starts to follow the same tendency than the case without interpolation. It can be noticed that when the graph passes from 60% of reference points to 50% the location error decreases from 2.16 m to 2.02 m. This improvement in the location error is due to the selection of the reference points. In some cases there are particular distributions in which the change in the selection of some few interpolated points can improve slightly the location estimation. The rationale behind this fact is that the location algorithm uses a set of candidate points with RSSI values concentrated in a small interval. Thus, in some cases the interpolated points replace some original misleading reference points and within this small degree of change in the database the average error can decrease slightly. In any case, this improvement is very small, lower than 10% of the location error that in the case of 60% of reference points. As a conclusion, this comparison demonstrates clearly the advantages of the interpolation to maintain the location accuracy when the number of reference points in the fingerprinting database is reduced.

Finally, just for the sake of comparison, two last experiments were carried out. In the first assessment, a comparison with the algorithm proposed in reference [7] is performed. The objective of this algorithm is to reduce the complexity of the database and the computation time decreasing the number of power levels and frequency channels used in the location, but it does not change the number of reference points. Thus, using the same experimental scenario presented in Section 5.1 with all the reference points and only the information that comes from one power level (P3) and three frequency channels (CH11, CH13, CH16), the algorithm achieves an average location error of 2.05 m. This result is practically the same that the one obtained in case A (with 50% reference points) using “Thin spline” interpolation, as it is shown in Table 2. So, it is concluded that with the interpolation the same location accuracy is achieved, but reducing significantly the initial effort to collect the reference points.

The second comparison is made introducing a threshold in the RSSI values saved in the database following the method proposed in the SIB algorithm [23]. The test was repeated with different thresholds, but with none of them the average localization error dropped below 2.5 m. The poor performance obtained with this variation of the location algorithm and its difference regarding the results presented in [23] might be due to: (a) the different size of both scenarios because the covered area in our case is smaller (19 × 11 m as opposed to the 52 × 48 m of SIB reference); (b) the number of beacons (4 in our case as opposed to 37); and (c) the power levels (4 in our case and 29 in the SIB reference). All these issues produce an important loss of information in our case that degrades the overall algorithm performance.

## 6. Conclusions

In this paper, we have proposed a method that reduces the training effort in RF fingerprinting location algorithms. The RSSI database built from the collected experimental measures includes only a reduced number of points. The rest of the reference points in the database get their values interpolating the samples from neighboring points. It is supposed that the algorithm relies on IEEE 802.15.4 networks, which allow the possibility of changing the frequency channel and radio power levels during a packet transmission. Using this feature, it is possible to collect RSSI values form multiple channels and power levels and combine all this information to increase the localization accuracy. The proposed localization algorithm is a variation of the KNN method that takes into account all the RSSI values form different channels and power levels. In the article, several experimental evaluations of this proposal are presented. Algorithms have been evaluated in a testing room with a testbed composed of four beacons and a grid of reference points. Initially, RSSI measures at every reference point were taken to build the training database. Then, a percentage of these points was removed and changed by new values generated using interpolation functions. Results demonstrate the validity of the method when the interpolated database is used, obtaining the same location accuracy that without interpolation. In the study, we have shown the influence of the selection and distribution of the reference points and proposed a non-uniform distribution of the reference points to improve this first selection process. Moreover, we have evaluated different interpolation functions, being the most adequate for our experiments the thin spline. Although the interpolation removing 50% of the initial points gives good results, it has been also proven that when the number of experimental points is further reduced (75% of interpolated points) the location performance is significantly degraded.

Finally, it is important to indicate that this work will be extended in the future to study the possibility of taking irregular distributions of points depending on the network beacons location. Additionally, an interesting approach will be to move the interpolation from the training step to the location step, which means that the database only contains the measured reference points and the interpolated points are computed only when they are needed on the location estimation. With this technique, the size of the database can be optimized and at the same time the effort in the training step is reduced.
